# Research Progress of Hydrogel Microneedles in Wound Management

**DOI:** 10.1021/acsbiomaterials.4c00972

**Published:** 2024-07-10

**Authors:** Ming Ji, Fangbiao Zhan, Xingan Qiu, Hong Liu, Xuezhe Liu, Pengzhen Bu, Bikun Zhou, Maciej Serda, Qian Feng

**Affiliations:** ‡ Department of Orthopedics, Chongqing University Three Gorges Hospital, School of Medicine, Chongqing University, Chongqing 404000, China; § Key Laboratory of Biorheological Science and Technology, Ministry of Educations, Collage of Bioengineering, Chongqing University, Chongqing 400044, China; ⊥ Institute of Chemistry, University of Silesia in Katowice, Katowice 40-006, Poland

**Keywords:** hydrogel microneedle, wound healing, drug delivery, micromolding, intelligentization

## Abstract

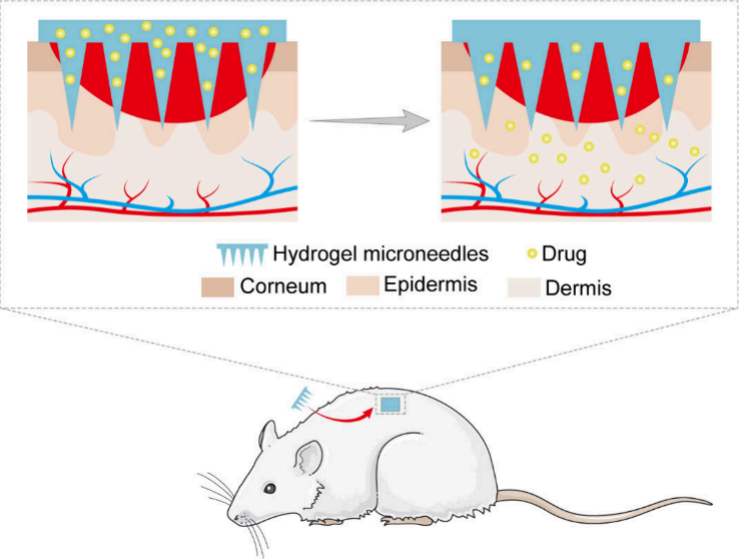

Microneedles are a novel drug delivery system that offers advantages such as safety, painlessness, minimally invasive administration, simplicity of use, and controllable drug delivery. As a type of polymer microneedle with a three-dimensional network structure, hydrogel microneedles (HMNs) possess excellent biocompatibility and biodegradability and encapsulate various therapeutic drugs while maintaining drug activity, thus attracting significant attention. Recently, they have been widely employed to promote wound healing and have demonstrated favorable therapeutic effects. Although there are reviews about HMNs, few of them focus on wound management. Herein, we present a comprehensive overview of the design and preparation methods of HMNs, with a particular emphasis on their application status in wound healing, including acute wound healing, infected wound healing, diabetic wound healing, and scarless wound healing. Finally, we examine the advantages and limitations of HMNs in wound management and provide suggestions for future research directions.

## 1 Introduction

As the largest organ of the human body, the skin plays a vital role as a defensive barrier, safeguarding the body against external harmful substances such as bacteria, viruses, and chemicals.^1^ However, external factors such as surgical procedures, external forces, heat, electricity, chemicals, low temperatures, and intrinsic factors like local blood supply disorders can damage the integrity of the skin and result in wound formation.^2,3^ Due to the regenerative properties of the skin, wounds typically heal rapidly under normal circumstances with only mild discomfort.^4^ However, certain factors, such as high blood glucose levels and local pressure, can significantly diminish the regenerative capacity of the skin.^5^ In these cases, prolonged exposure of the wound may result in infection or other complications, further impeding wound healing. In 2014, over 8 million individuals in the United States were affected by wounds, with estimated losses reaching 30 billion dollars.^6^ Notably, chronic skin wounds such as pressure ulcers and diabetic foot ulcers have shown a steady upward trend, now impacting more than 1% of the population throughout their lifetime.^7^ Given the aging of the population, diabetes, obesity, and persistent problem of infection, it is expected that chronic wounds will continue to pose a severe challenge to the world’s health systems, resulting in tremendous economic losses.^8,9^ Therefore, wound treatment, especially for chronic wounds, has been a focal point of research in the medical field.

The treatment of chronic wounds typically involves the delivery of drugs or cytokines to eliminate bacterial infection and regulate the microenvironment of the wound area, thereby promoting wound closure.^10,11^ However, while the presence of blood clots and scar tissue serves as barriers for wounds, they also impede the effective delivery of drug molecules to the targeted area.^12^ Furthermore, in certain wound types, the persistent exudation of wound fluid can wash away therapeutic drugs from the wound bed.^13^ Consequently, the bioavailability of administered drugs for wound treatment often falls below anticipated levels.^14^ Therefore, achieving optimal outcomes in wound therapy necessitates the development of more precise and efficient approaches for local drug administration.

Microneedle technology is a novel technique that has garnered significant attention from researchers in the field of transdermal drug delivery.^15^ It involves the use of submillimeter-scale microneedle arrays (MNAs) to penetrate the skin without contacting nerves or blood vessels, enabling minimally invasive and painless drug administration.^16^ Due to the unique structure of microneedles, they exhibit distinct advantages in wound healing and tissue regeneration: (1) Microneedles can overcome physical barriers, such as clots, scars, and exudates, allowing for sustained drug release. (2) The length of microneedles does not reach the nerve endings, offering a painless delivery method that enhances patient compliance.^16^ (3) Microneedle administration allows for precise drug dosing, ensuring accurate drug delivery and minimizing toxic side effects.^17^ (4) The needle tips of microneedles can breach bacterial biofilms, facilitating the release of antimicrobial drugs into the interior of the biofilm through the interstitial fluid (ISF) at the wound site.^18^

**Figure 1:**
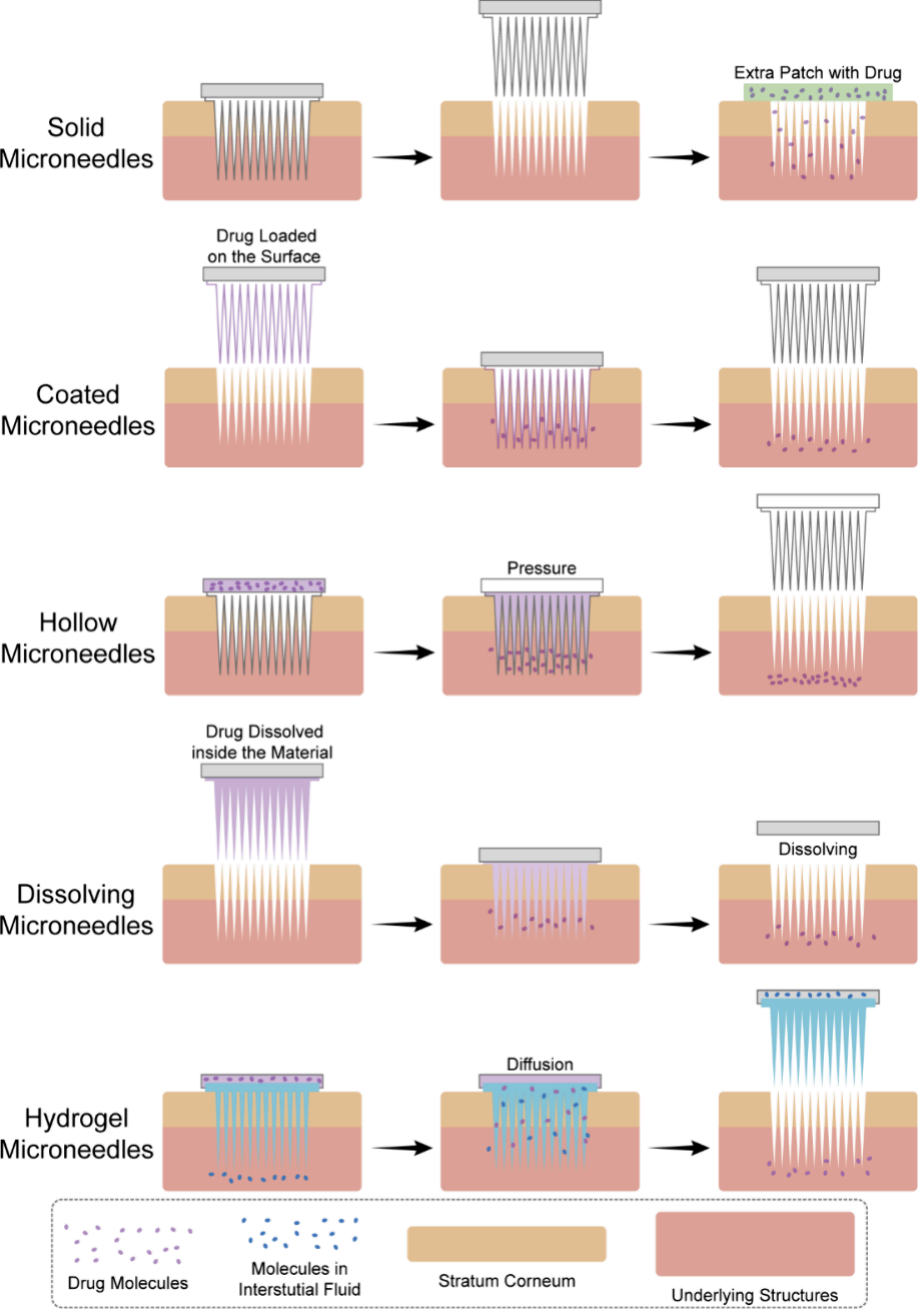
Schematic diagram illustrating the mechanisms involved in transdermal drug delivery and ISF extraction (HMNs only) utilizing various types of microneedles.

Based on the drug types and administration methods, researchers have developed various types of microneedles, including solid microneedles,^19^ coated microneedles,^20^ hollow microneedles,^21^ dissolving microneedles,^22^ and hydrogel microneedles (HMNs).^23^ The structures of these microneedles and their mechanisms of drug release or ISF extraction (HMNs only) are illustrated in Figure 1. Solid microneedles are applied by puncturing the skin and attaching drug patches, but the process is cumbersome.^24,25^ Coated microneedles have drug coatings on the surface of solid microneedles, allowing drugs to diffuse into deeper epidermal layers.^26^ However, due to limited coating thickness, they cannot deliver high doses of drugs. Hollow microneedles, similar to short subcutaneous injection needles, can precisely deliver high doses of drugs, but they have lower mechanical strength, and repeated use may lead to blockage and infection.^27^ Dissolving microneedles are made of water-soluble polymers, exhibiting good biocompatibility and eliminating the need for needle removal.^28,29^ However, dissolving microneedles often show low mechanical strength, making it difficult to be inserted into the skin, and they require time to dissolve, resulting in delayed drug delivery. Additionally, long-term use of dissolving microneedles may lead to polymer distribution and deposition throughout the body, causing issues such as immune rejection.^30^ HMNs were first proposed by Donnelly et al.^23^ in 2012, who used poly(methyl vinyl ether-*co*-maleic acid) (PMVE/MA) and ethylene glycol to prepare HMNs for insulin delivery. HMNs are prepared from cross-linked polymers and, upon insertion into the skin, form continuous and unobstructed drug delivery channels by absorbing ISF.^31^ Compared to other microneedles, HMNs offer good flexibility, can be fabricated into various shapes, and are easy to peel off from the skin without residue.^32^Table 1 summarizes the advantages and disadvantages of different types of microneedles.

**Table 1:** Comparison of Advantages and Disadvantages of Different Microneedles

	Solid microneedle	Coated microneedle	Hollow microneedle	Dissolving microneedle	Hydrogel microneedle
Drug loading dose	High	Low	High	High	High
Drug release rate	Uncontrollable	Uncontrollable	Controllable	Uncontrollable	Controllable
Mechanical strength	High	Relatively high	Low	Low	Relatively high
Risk of occlusion	High	Relatively high	High	Low	Low
Drug delivery steps	Complex	Simple	Simple	Simple	Simple
Preparation process	Simple	Complex	Complex	Simple	Relatively simple

In recent years, HMNs have gained significant traction across various domains, including drug delivery,^33,34^ vaccine administration,^35^ minimally invasive extraction,^36,37^ and cancer treatment,^38,39^ due to their distinctive advantages. While existing literature provides reviews on the broad applications and potential benefits of HMNs,^40,41^ there is currently a dearth of comprehensive reviews specifically focusing on the utilization of HMNs in wound treatment. To address this gap, we have undertaken a meticulous review encompassing HMN-assisted wound healing. In this review, we present an overview of the design and fabrication methods employed in the development of HMNs. Additionally, we critically evaluate their potential applications in wound treatment. Furthermore, we analyze the advantages and limitations associated with HMNs in the context of wound healing, and offer valuable insights into their future advancements. This review aims to provide clinicians and researchers in the field of wound treatment with valuable guidance and perspectives, thereby fostering progress in this area of study.

## 2 Design of HMNs

**Figure 2:**
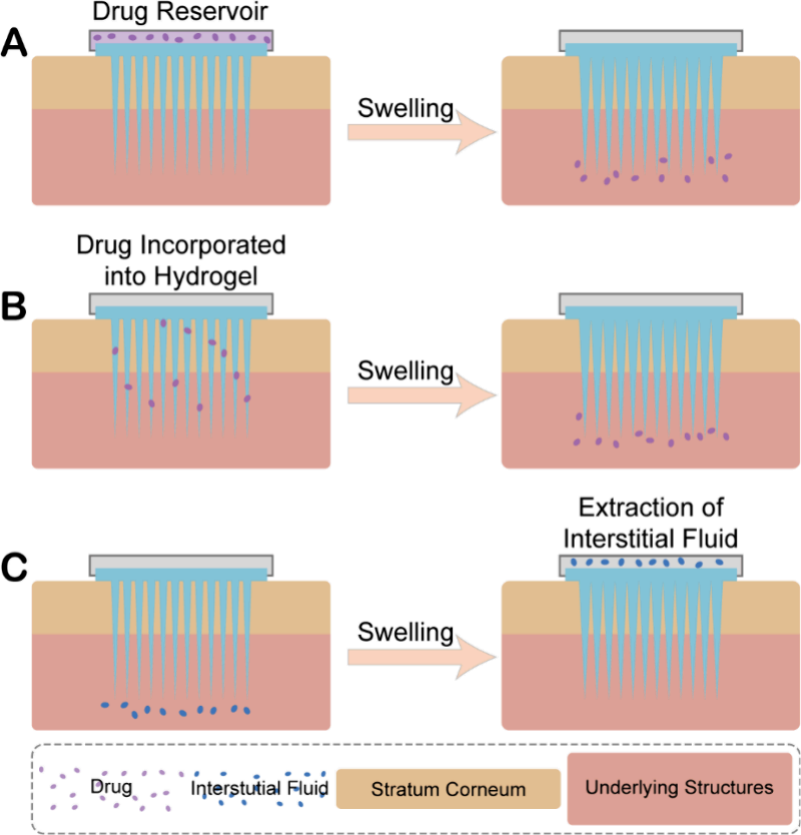
Mechanism of drug administration via (A) drug reservoir or (B) incorporated drug and (C) ISF extraction after swelling of HMNs.

HMNs should possess sufficient mechanical strength in the dried state to allow for insertion into the epidermis. Upon insertion, they absorb ISF and undergo a swelling transition to a gel state facilitated by the three-dimensional network structure of the hydrogel. This unique property enables HMNs to function as a minimally invasive diagnostic method, as the absorbed ISF can provide valuable biomarkers. Moreover, the hydrogel network facilitates the formation of continuous channels, allowing drugs to diffuse into deeper layers of the skin along concentration gradients.^32^ This characteristic highlights the remarkable potential of HMNs in transdermal drug delivery. The swelling capacity of HMNs, which governs the drug release rate and loading amount, can be modulated by adjusting the cross-linking density of the three-dimensional network structure.^42,43^ Drugs within HMNs can be stored in needle tips, microneedle arrays, or separate drug reservoir patches. The drug reservoir patch is positioned at the back of HMNs and diffuses into the skin through the continuous channels formed by the absorption of ISF by HMNs. The drug being delivered can be altered by replacing the drug reservoir patch.^44^Figure 2 illustrates the operational mechanism of drug delivery and ISF extraction subsequent to the swelling of HMNs.

### 2.1 Matrix Materials of HMNs

The performance of HMNs is influenced by the matrix materials. The materials that form the hydrogel include natural polymers and synthetic polymers. Currently, the materials used for preparing HMNs mainly include PMVE/MA, poly(vinyl alcohol) (PVA), methacrylated hyaluronic acid (MeHA), silk fibroin (SF), Gelatin Methacryloyl (GelMA), sodium alginate (SA), and chitosan (CS), etc.

#### 2.1.1 Poly(methyl vinyl ether-*co*-maleic acid) (PMVE/MA)

PMVE/MA, a synthetic copolymer with a mass ratio of 1:1 between poly(methyl vinyl ether) and maleic acid, exhibits excellent biocompatibility and low toxicity, making it a suitable biomaterial for HMN applications.^45^ For example, Chandran et al.^46^ developed a HMN system for transdermal caffeine delivery, providing a viable solution for caffeine transdermal administration. The system consisted of PMVE/MA copolymer and varying concentrations of sodium bicarbonate. Through their study, it was found that HMNs made from PMVE/MA copolymer with 3% mass fraction of sodium bicarbonate exhibited optimal physical and swelling properties, improving the transdermal delivery of the hydrophilic drug, caffeine. D’Amico et al.^47^ developed a bilayered microneedle patch using polyvinylpyrrolidone (PVP) and PMVE/MA polymer. The microneedle patch showed an initial burst release followed by sustained release over several hours. Compared to the commercial oral formulation of meloxicam, the microneedle patch improved the in vivo bioavailability of the drug.

To enhance the swelling rate and mechanical properties of PMVE/MA hydrogels, cross-linking with different cross-linkers such as polyethylene glycol (PEG), glycerol, pectin, etc., is commonly employed. PEG is the most widely used cross-linker. For instance, Romanyuk et al.^48^ prepared HMN patches by cross-linking a 15% mass fraction of PMVE/MA polymer with a 7.5% mass fraction of PEG for collecting ISF from rat skin. The patch exhibited good swelling properties, with a maximum swelling ratio exceeding 5000%. Migdadi et al.^33^ developed a drug reservoir-type HMN composed of PMVE/MA polymer cross-linked with PEG. The microneedles consisted of a drug reservoir layer and a microneedle layer, with the microneedle layer formed by esterification cross-linking of 20% mass fraction of PMVE/MA with 7.5% mass fraction of PEG. Mechanical strength experiments demonstrated that the microneedles had sufficient mechanical strength. Al-Badry et al.^49^ developed a HMN prepared from PMVE/MA copolymer cross-linked with PEG for transdermal delivery of acyclovir. Experimental results showed that the microneedles successfully penetrated the skin barrier and delivered acyclovir in a sustained manner over 24 h.

#### 2.1.2 Poly(vinyl alcohol) (PVA)

PVA is a water-soluble polymer that exhibits excellent water swelling, biocompatibility, and nontoxic properties, making it highly promising for applications in the biomedical field.^50^ PVA has a strong ability to form cross-linked structures without the need for toxic cross-linkers. Additionally, due to its slow swelling rate, PVA-based HMNs enable sustained drug release.^51^

In the preparation of PVA-based HMNs, the addition of different polymers is often employed to enhance the toughness, mechanical strength, and porosity of PVA hydrogels, thereby expanding the medical applications of PVA HMNs. For example, He et al.^37^ incorporated CS into PVA to prepare PVA/CS HMNs for ISF extraction. The addition of CS significantly improved the mechanical strength, porosity, and water absorption of the PVA/CS hydrogel. Xu et al.^52^ introduced PVP into PVA to fabricate PVA/PVP HMN patches for ISF extraction. By utilizing mild heating to extract ISF due to the thermal degradation of PVA, the accuracy of detection was greatly enhanced. The HMN was capable of insertion into the skin in a dry state and exhibited good swelling properties, enabling ISF extraction within a short period. Fitri et al.^53^ prepared PVA/PVP HMNs for the delivery of sildenafil citrate to treat pulmonary arterial hypertension, successfully incorporating sildenafil citrate into a system of HMN and tablet reservoir, with the potential to enhance the therapeutic effect of pulmonary arterial hypertension. Aziz et al.^54^ developed PVA-based HMNs for transdermal delivery of albendazole. The microneedles exhibited high swelling capacity exceeding 400% and a penetration rate of 63%. The cumulative amount of albendazole permeated into the skin was approximately (971.23 ± 11.77) μg/cm^2^, offering a potential solution to overcome the limitations of albendazole in oral formulations. Aung et al.^55^ incorporated poly(acrylic acid) -*co*-maleic acid (PAMA) into PVA to prepare PAMA/PVA HMNs for delivering α-arbutin to the skin. In vivo permeation studies and in vivo experiments demonstrated that PAMA/PVA HMNs provided high intradermal delivery of α-arbutin levels, surpassing that of gels and commercial creams. Furthermore, the micropores of skin induced by HMNs resealed within 1 h. Hasnain et al.^56^ developed arginine-modified CS/PVA HMNs as a transdermal delivery system for curcumin to promote wound healing and enhance antimicrobial activity. The microneedles exhibited excellent swelling capacity, enabling the absorption of wound exudate and controlled drug release, thereby accelerating wound healing.

#### 2.1.3 Methacrylated Hyaluronic Acid (MeHA)

Hyaluronic acid (HA) is an acidic, viscous polysaccharide that possesses excellent biocompatibility, biodegradability, and chemical modifiability, making it widely utilized in the field of biomedical research.^57^ Due to the enzymatic degradation of HA by hyaluronidase, its half-life in the body is relatively short. After modification with methacrylic acid ester groups, HA can be cross-linked via photopolymerization to form MeHA.

Compared to HA hydrogels, MeHA hydrogels exhibit higher stability and mechanical properties while retaining excellent biocompatibility, making them commonly employed in the fabrication of HMNs.^58^ For instance, Chang et al.^36^ utilized MeHA to prepare a HMN patch capable of rapid ISF extraction. This HMN patch enabled rapid ISF extraction without the need for additional devices. Furthermore, it maintained structural integrity after use, avoiding residue within the skin. GhavamiNejad et al.^59^ developed an intelligent composite HMN patch using MeHA MNAs and embedded multifunctional microgels that released native glucagon at low glucose levels. In type 1 diabetic rats, the transdermal application of this patch prevented hypoglycemia, demonstrating promising applications. Chew et al.^60^ introduced a MeHA HMN system capable of effectively loading and demonstrating burst release of diverse therapeutics, including small hydrophilic molecules, hydrophobic compounds, and biomacromolecules. Qin et al.^61^ fabricated antimicrobial and angiogenesis-promoting HMNs for wound dressing using MeHA in combination with dimethyloxalylglycine, pH-responsive functionalized zeolitic imidazolate framework-8 (ZIF-8) nanoparticles (NPs). This therapeutic approach successfully accelerated the healing of infected wounds in rats.

#### 2.1.4 Silk Fibroin (SF)

SF is a natural high molecular weight fibrous protein extracted from silk through a degumming process.^62^ Possessing excellent flexibility, ductility, biocompatibility, biodegradability, and mechanical properties, SF is also susceptible to chemical modifications, making it an ideal material for the fabrication of microneedles.^63^

Despite its many advantages, pure SF microneedles are soluble in water,^64^ necessitating modifications such as 2-ethoxyethanol, semi-interpenetrating network (semi-IPN), CS, and methacryloyl groups to prepare water-insoluble microneedles for sustained drug release. For example, Yin et al.^64^ created a composite material by mixing 2-ethoxyethanol with SF and fabricated microneedles with controlled release mechanisms and excellent biocompatibility, offering prospects as a viable transdermal delivery system. Chen et al.^65^ innovatively developed a glucose-responsive microneedle by combining SF with a semi-IPN hydrogel of phenylboronic acid/acrylamide, specifically designed for insulin delivery. To enhance its mechanical properties, they cleverly designed a double-layer microneedle structure: the upper needle region consisted of a semi-IPN hydrogel containing SF, while the lower layer was composed entirely of SF. This design not only increased the structural strength of the microneedles but also enabled rapid response to changes in blood glucose levels, facilitating on-demand insulin release. Jia et al.^66^ utilized grafting techniques of CS onto SF to successfully develop a positively charged SF/CS HMN. This microneedle demonstrated excellent mechanical performance and exhibited pH-responsive swelling properties. Results from in vitro transdermal drug release experiments revealed that the release behavior of insulin loaded within the microneedles was significantly influenced by changes in pH, with a faster release rate observed in acidic environments compared to neutral conditions. Therefore, by flexibly adjusting the pH of the solution, the microneedles can sensitively respond to external environmental changes, achieving intelligent control of transdermal insulin release. Sun et al.^67^ designed an oxygen-generating SilMA-based microneedle for the treatment of diabetic wounds. Its tips were encapsulated with calcium peroxide and catalase, allowing sustained oxygen release and suppression of reactive oxygen species (ROS). Additionally, the bottom of the patch was coated with antibacterial AgNPs to effectively combat microbial infections and further promote wound healing.

#### 2.1.5 Gelatin Methacryloyl (GelMA)

GelMA, as a gelatin derivative, is formed by cross-linking gelatin with methacrylic anhydride under the assistance of a photoinitiator through ultraviolet or visible light.^68^ This material has been widely studied and applied in the field of bioengineering and biomedicine due to its high biocompatibility and excellent drug delivery performance.^69^

GelMA possesses several advantages, such as tunability, low immunogenicity, and sufficient mechanical strength for skin penetration, making it commonly used for the fabrication of HMNs. For example, Guo et al.^70^ developed a glucose-responsive insulin-releasing HMN patch for the treatment of diabetic wounds. The HMN patch consisted of GelMA, glucose-responsive monomer 4-(2-acrylamidoethylcarbamoyl)-3-fluorophenylboronic acid, and gluconic insulin. It exhibited adequate mechanical properties, high biocompatibility, insulin release behavior responsive to different glucose solutions, and strong adhesion to the skin. Haghniaz et al.^71^ developed a biocompatible and biodegradable HMN based on GelMA for hemorrhage control, achieved through hybridization with silicate nanosheets. The MNAs with silicate nanosheets imparted hemostatic functionality, while the needle-shaped structures increased the contact area with blood, resulting in a significant reduction of clotting time from 11.5 to 1.3 min in vitro. In a rat liver bleeding model, the GelMA-based HMN reduced bleeding volume by approximately 92% compared to the untreated bleeding group. Zhao et al.^72^ utilized a separable microneedle enriched with *Chlorella vulgaris* for controlled oxygen delivery to facilitate diabetic wound healing. The microneedle was composed of a PVA substrate and GelMA tips encapsulating *Chlorella vulgaris*. When applied to diabetic wounds, the PVA substrate rapidly dissolved within a short period, while the nontoxic and biocompatible GelMA tips remained in the skin. Through the photosynthetic activity of *Chlorella vulgaris* the microneedle was able to sustainably generate oxygen in a green manner and release it in a controlled manner, effectively promoting cell proliferation, migration, and vascularization. This approach significantly enhanced wound healing in diabetic mice. Chen et al.^73^ developed a multifunctional drug delivery system based on ZIF-8 and GelMA aiming for stable, transdural, and controlled sustained release of drugs in spinal cord injury treatment. The system utilized HMN to create microscale pores on the dura mater for direct delivery of methylprednisolone sodium succinate to the spinal cord. The combination of ZIF-8 and GelMA HMN extended the release period of methylprednisolone sodium succinate up to 5 days. Yang et al.^74^ proposed a novel detachable HMN composed of photopolymerized GelMA and 5-FuA-Pep-MA prodrug, which could respond to the elevated levels of ROS and overexpression of matrix metalloproteinases in hypertrophic scar pathological microenvironment. In vivo experiments on female mice demonstrated sustained drug release achieved by the retention of the microneedle tips in the tissue. Importantly, the drug-loaded microneedles were able to reshape the pathological microenvironment of hypertrophic scar tissue in female rabbit ears by clearing ROS and depleting matrix metalloproteinases.

#### 2.1.6 Sodium Alginate (SA)

SA is a naturally occurring anionic macromolecule that possesses multiple advantages, including nontoxicity, abundant source, biodegradability, renewability, and excellent biocompatibility. It can be combined with other functional components through physical or chemical cross-linking.^75^ When SA (monovalent ions) undergoes an exchange with calcium ions (divalent ions), the original low-viscosity solution rapidly transforms into a gel structure.^76^

HMNs prepared using SA exhibite excellent mechanical properties, biocompatibility, and swelling behavior after cross-linking, making them stand out in the field of transdermal drug delivery. For example, Zhang et al.^77^ utilized a template method to fabricate calcium ion-cross-linked alginate/maltose (Ca^2+^/Alg-Mal) composite microneedles. These microneedles demonstrated significant mechanical strength, with a maximum fracture force of 0.41 N/needle. Due to their outstanding mechanical properties and biocompatibility, the prepared Ca^2+^/Alg-Mal composite microneedles have been successfully applied for transdermal insulin delivery in diabetic Sprague–Dawley rat models, showing comparable relative pharmacological utilization and relative bioavailability to the subcutaneous injection route. This offered a new potential strategy for the treatment of diabetes, with the prospect of reducing the inconvenience and pain associated with traditional injection methods. Zhou et al.^78^ explored different manufacturing methods and successfully constructed a flat-based HMN based on alginate in situ hydrogel, with its gelation process driven by ethylenediaminetetraacetic acid calcium disodium salt and D-(+)-glucono-1,5-lactone. Further research results indicated that this HMN exhibited excellent mechanical properties and biocompatibility, potentially broadening the range of drugs that can be used for transdermal delivery. Shan et al.^79^ developed a a bifunctional double-layer microneedle platform that combines both chemo-photothermal synergistic melanoma treatment and skin regeneration. The outer layer consisted of dissolvable microneedles, while the inner layer was composed of a nondissolvable SA/gelatin/HA as the supporting backing layer. Once the embeddable microneedles were inserted into the skin, they rapidly dissolved and successfully activated drug release for tumor treatment. Meanwhile, the SA/gelatin/HA supporting backing layer remained on the wound surface, covering the injury and promoting the proliferation of endothelial cells and fibroblasts, thereby accelerating skin regeneration. This double-layer microneedle platform combined the advantages of chemotherapy and photothermal therapy, enabling the elimination of tumors and accelerated wound healing simultaneously. It held promise as a competitive strategy for the treatment of melanoma.

#### 2.1.7 Chitosan (CS)

CS is a semisynthetic cationic linear polysaccharide synthesized by the deacetylation of natural polysaccharide chitin. It possesses nontoxicity, high biocompatibility, biodegradability, low immunogenicity, and natural antimicrobial properties, making it highly suitable for biomedical applications.^80^ CS can form a gel without the need for external cross-linking agents and is commonly used in the fabrication of HMNs. For instance, Chi et al.^81^ utilized CS to prepare HMN patches through physical cross-linking and applied them for wound healing. Further studies demonstrated that the HMN patches could suppress inflammatory reactions during the wound closure process and promote collagen deposition, vascularization, and tissue regeneration. Dathathri et al.^82^ successfully prepared CS/PVA HMN by mixing CS with PVA and drying them in molds. Subsequent research revealed that effective cross-linking occurred between CS and PVA, enabling the prepared CS/PVA HMN to achieve sustained drug release of 20.17% over a period of 30 h, offering new possibilities for painless and sustained transdermal drug delivery. Dai et al.^83^ developed a safe and effective HMN patch utilizing methacryloyl-modified CS (CSMA) as a continuous drug delivery platform for the treatment of psoriasis. By systematically optimizing the preparation process of CSMA, they successfully fabricated well-shaped and mechanically robust (0.7 N/needle) CSMA HMNs with a CSMA concentration of only 3% (w/v). Further research demonstrated that the HMN patch achieved 80% sustained drug release within 24 h in vitro and effectively suppressed skin thickening and splenomegaly in psoriasis mice, exhibiting good biocompatibility at adequate therapeutic dosages.

### 2.2 Geometric Parameters

The geometric parameters of HMNs, including shape, length, spacing, and needle tip diameter, are crucial to their application effectiveness. When designing HMNs, specific parameters must be considered to ensure a balance between the array and size of HMNs and their swelling capacity.

A variety of HMN shapes have been reported, including pyramid-shaped,^36^ conical,^81^ bullet-shaped,^84^ eagle claw-shaped,^85^ shark tooth-shaped,^86^ pagoda-shaped,^87^ and others, with pyramid-shaped and conical HMNs receiving particular attention. Study has shown that compared to conical HMNs, pyramid-shaped microneedles exhibit superior mechanical strength, attributed to their larger cross-sectional area with the same base width, thereby enhancing structural stability.^88^

The ideal length of microneedles should range from 25 to 2000 μm, ensuring penetration through the stratum corneum for drug delivery while avoiding contact with the neural layer of the dermis to minimize pain.^89^ Increasing the length of microneedles enhances the sensation of pain while reducing mechanical strength. The impact of microneedles on the barrier function of human skin was found to be greater for 600 μm microneedles compared to those measuring 400 and 1000 μm, as observed through TEWL measurements.^90^ This indicated that the influence of microneedles on the barrier function of the skin did not increase indefinitely with their length but rather reaches a specific threshold. Researchers evaluated the transdermal drug delivery capability of a series of microneedles ranging from 100 to 1100 μm and found that 600 μm was the optimal length.^91^ Beyond this length, further increases in microneedle length did not significantly improve drug delivery. Therefore, in microneedle design, a comprehensive consideration of the relationship between length, pain, mechanical strength, and barrier function of the skin is necessary to achieve optimal drug delivery efficacy.

When applying microneedle technology in clinical settings, precise control over the depth of insertion into the skin is necessary. If the tip diameter is too large, significant force is required to penetrate the skin. It was reported that microneedles with smaller tip diameters exhibit smoother skin penetration, and as the insertion force increased, their penetration depth showed a more linear trend.^92^

Microneedle density is another important design parameter. With increasing microneedle density, both the degree of impact on the skin barrier function and the amount of drug delivery are expected to increase. However, once the microneedle density reached a certain threshold, further increasing the density did not significantly enhance the impact on the skin barrier function or the amount of drug delivery.^90,91^ This might be due to the occurrence of a “nail bed effect”. Therefore, to avoid the “nail bed effect”, researchers tend to favor using MNAs with a “low needle density”, approximately 220 needles per square centimeter.^32^

### 2.3 Intelligentization

The delivery of drugs by HMNs is generally passive transportation, which cannot control the drug release process.^14^ Introducing stimuli-responsive materials to prepare intelligent responsive HMNs holds promise for addressing this issue. Stimuli-responsive materials enable the structure and functionality of HMNs to respond to external stimuli, including temperature, pH, light, electric field, magnetic field, enzyme, or ion concentration, etc.^93^ Therefore, intelligent responsive HMNs can intelligently regulate the drug release rate according to the severity of the disease, potentially enhancing the therapeutic effect. For example, Hardy et al.^94^ reported a stimuli-responsive HMN array capable of delivering the model drug ibuprofen under light stimulation. The array was prepared using a specific polymer micromolding method, exhibiting excellent mechanical properties and the ability to load high-concentration drugs. In vitro experiments demonstrated that the system could deliver drugs for a long time and multiple times, showing potential as a controlled release device. This technology was expected to be used in various application scenarios to achieve “on-demand” delivery of multiple drugs and enhance patient care. Bi et al.^95^ designed detachable ROS-responsive HMN patches containing methotrexate and epigallocatechin-3-gallate, achieving dual-mode drug release, with rapid release of antiproliferative drugs and sustained responsive release of anti-inflammatory drugs. This intelligent microneedle system significantly prolonged the drug residence time in the skin and improved the therapeutic effect, demonstrating promising application prospects in psoriasis-like animal models. Zhu et al.^96^ prepared HMNs with dual-mode controlled drug delivery functionality by mimicking the teeth and venom secretion of blue-ringed octopus. Within the first 2–4 h of treatment, the microneedles sensed the body temperature and actively injected a portion of the drug into the tissue to achieve a rapid therapeutic effect for the disease treatment.

## 3 Fabrication Methods of HMNs

The HMNs can be obtained by injecting hydrogel polymers into microneedle molds using specific methods and subsequently removing them from the molds after drying. Various techniques, including micromolding, 3D printing, and in situ formation, are commonly employed in the preparation of HMNs. The selection of these fabrication methods typically depends on the choice of matrix materials and cross-linking approaches utilized.

### 3.1 Micromolding

**Figure 3:**
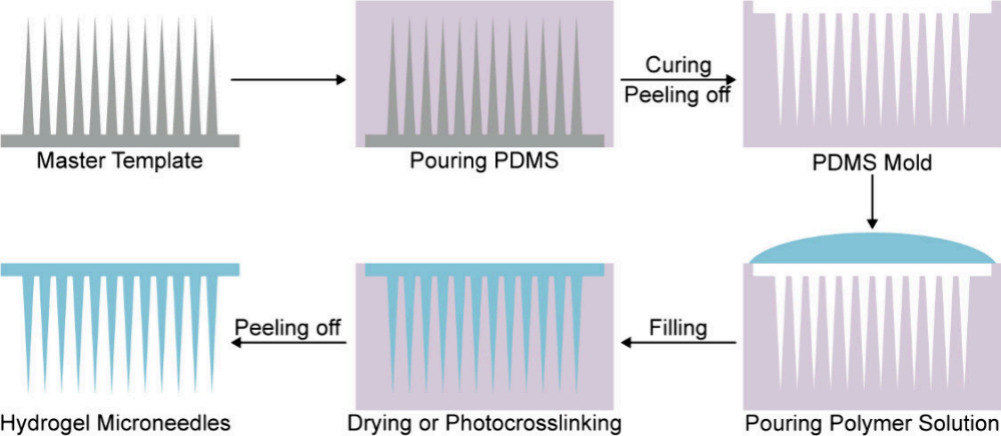
Schematic diagram of HMNs fabricated via PDMS micromolding.

Currently, micromolding has become the mainstream technology for preparing HMNs. In the process of mold preparation, polydimethylsiloxane (PDMS), a flexible hydrophobic material, is widely used to manufacture intricate microneedle structures.^97^ In the preparation of PDMS microneedle templates, the pouring method is commonly employed, which involves pouring a mixture of PDMS prepolymer and curing agent onto the surface of a preformed solid microneedle template. After the mixture solidifies, a microneedle template with the desired structure is obtained. Subsequently, the polymer solution is filled into the microneedle cavities, ensuring full penetration of the solution into the microneedles through vacuum or centrifugation. Upon drying or photo-cross-linking, the microneedles can be demolded to obtain the desired structure (Figure 3). PDMS molds exhibit excellent replication performance and can be easily replicated in large quantities from a single master template to meet the demands of large-scale production.^43^ Currently, the application of micromolding in the preparation of HMNs has been extensively reported. For example, Ye et al.^98^ successfully prepared HMN microneedles using the micromolding technique. The innovation of those microneedles lay in their utilization of the dynamic covalent interaction between phenylboronic acid and diol bonds, which not only achieved network cross-linking but also imparted glucose-responsive characteristics. The research results demonstrated that under the influence of glucose, the release rate of insulin from the microneedles was accelerated, effectively controlling the short-term blood glucose levels in a diabetic rat model. Zeng et al.^99^ successfully prepared composite HMN microneedles using PDMS molds and micromolding. Those microneedles primarily consisted of HA as the matrix material and were loaded with dexamethasone for the treatment of oral ulcers. Experimental results showed that those composite HMNs could precisely deliver therapeutic drugs to the lesion site within a short period of time. Furthermore, the microneedles had the ability to simultaneously load multiple drugs, which not only reduced the risk of infection but also accelerated the wound healing process.

Although the pouring method is a cost-effective and simple technique for preparing microneedles that accurately replicate the overall architecture of the microneedle master, it does not allow for fine adjustment and optimization of the microstructural features of the microneedle template. To address this limitation, researchers have conducted various studies. For example, Chen et al.^100^ proposed a method that involved introducing a metal microneedle cannula, and by adjusting the pouring volume of PDMS solution inside the cannula, the length of the microneedles could be modified. This system demonstrated controllable microneedle length and uniform distribution of the needles, providing a low-cost and effective method for microneedle fabrication. Additionally, there have been research that directly carved microhole structures on PDMS materials using laser ablation.^101^ The advantages of laser-prepared microneedle templates included the ability to design and optimize structures during the fabrication process, allowing for the production of PDMS microneedle molds with virtually any height and spacing.

Micromolding is a method for preparing HMNs by molding and curing polymers in a mold to achieve a hydrogel state. Since the mold can be reused, it enables convenient and rapid production of multiple HMNs. This method facilitates parameter optimization, exhibits good reproducibility, and is suitable for large-scale production. However, it should be noted that the preparation process may compromise the activity of certain drugs, particularly sensitive drugs such as proteins and vaccines.^102^

### 3.2 3D Printing

3D printing technology is an advanced emerging technology that is based on three-dimensional computer-aided design models.^103^ It enables rapid prototyping of various complex three-dimensional objects by layer-by-layer printing and continuous stacking of adhesive materials. Compared to micromolding, 3D printing technology has the advantages of directly printing microneedles into any desired shape and achieving customized production of microneedles with various parameters.^104^

Due to its ability to achieve precise control over microneedle shape and various parameters, 3D printing has emerged as a highly attractive method for HMN preparation. For instance, Yao et al.^105^ proposed the fabrication of HMNs using a high-precision digital light processing 3D printing system capable of performing multiple tasks such as drug delivery and sensing. By optimizing printing parameters, the microneedles achieved a balance between accuracy and stiffness while exhibiting excellent biocompatibility. This low-cost and rapid approach provided strong support for microneedle construction and potential clinical applications. Cordeiro et al.^103^ employed two-photon polymerization 3D printing to fabricate a series of master templates that could be used to produce multiple MNA molds for HMN preparation. The HMNs prepared using this method demonstrated satisfactory results in terms of insertion, drug loading, and transdermal drug delivery. Barnum et al.^106^ developed a low-cost, simple, yet robust strategy for MNAs fabrication using 3D printing technology. These MNAs consisted of a rigid resin-based outer layer and a bioactive molecule-encapsulating SA hydrogel inner layer. Further research demonstrated its capability to encapsulate and subsequently deliver vascular endothelial growth factor (VEGF). This study opened up new research directions for the transdermal delivery of bioactive molecules using 3D-printed MNAs. Inspired by the teeth and venom secretion of blue-ringed octopus, Zhu et al.^96^ combined high-precision 3D printing with biomimetic suction to develop an actively injectable HMN that ensured on-demand drug release. Additionally, the biomimetic suction ensured secure fixation of the microneedles in wet environments. This microneedle patch combined wet adhesion and multimodal delivery, accelerating ulcer healing and inhibiting tumor progression, providing a new approach for localized therapy.

3D printing offers significant advantages in the manufacturing of HMNs, facilitating direct design, modification, and fabrication of microneedles with precise dimensional features. This simplifies the design and prototyping process, enabling quick responses to evolving demands and cost reduction. However, 3D printing does have limitations, such as relatively slow printing speeds, limited resolution, and a constrained selection of materials, which somewhat restrict its application range.^107^ Moreover, challenges pertaining to biocompatibility and mechanical performance must be addressed.^107^ Nevertheless, continuous technological advancements are expected to broaden and deepen the application of 3D printing in the field of microneedle manufacturing.

### 3.3 In Situ Formation

In-situ formation technology is an innovative approach for preparing HMNs by inducing the sol–gel transition of polymer sols. The process involves the insertion of solid microneedles into the skin to create microchannels, followed by the application of a drug-loaded polymer formulation onto the microchannels. Subsequently, the polymer undergoes a transformation within the skin, resulting in the formation of HMNs that enable sustained drug delivery. This process maximizes the utilization of the sol–gel transition to ensure continuous drug efficacy at the intended site. For instance, Sivaraman et al.^108^ utilized a biocompatible nonionic thermoresponsive copolymer to fabricate in situ forming HMNs for transdermal drug delivery. By leveraging the sol–gel transition properties of poloxamer, the researchers successfully prepared and evaluated microneedles for drug delivery. Experimental results demonstrated stable and sustained delivery of methotrexate using the microneedles on porcine ear and human skin. The in situ forming HMNs represented a novel and effective approach to transdermal drug delivery, with potential clinical applications.

Due to the specific material requirements of in situ formation technology, there is currently limited research employing this method.

## 4 Applications of HMNs in Wound Healing

Wound healing, especially the healing of complex wounds, has always been a challenging task in clinical practice. In recent years, an increasing number of studies have focused on exploring the use of HMNs to assist in wound healing. This is due to the unique advantages of HMNs, including good biocompatibility, strong drug-carrying capacity, convenient administration, and minimal side effects. This section summarizes the outstanding work accomplished in the past five years regarding the use of HMNs for assisting in wound healing, providing key reference cases for designing relevant treatment strategies (Table 2).

**Table 2:** Summary of HMNs for Wound Healing^a^

Application	Materials	Fabrication method	Function	Ref
*Acute wound healing*	Mussel adhesive protein/HA/SF	Micromolding	Wound sealing	(110)
	Gauze/liquid metal/PEGDA	Micromolding	Wound sealing/ES	(85)
	PLGA/GelMA	Micromolding	Delivery of MSCs	(111)
	carbon nanotube/HAMA/PEGDA	Micromolding	Delivery of VEGF/fibroblast orientation	(112)
	SF/PU/NIPAM/SiO_2_ NPs	Micromolding	Delivery of VEGF and mupirocin	(150)
	SP/aloe vera gel/PU/EGaIn	3D printing	Delivery of VEGF	(151)
	MXene/SF/PU	Micromolding	Delivery of hEGF	(113)
	MXene/PEGDA/PBA	Micromolding	Delivery of adenosine	(114)
	CS/PVA	Micromolding	Delivery of asiatic acid	(115)
*Infected wound healing*	PEGDA/acrylamide/alginate/triboelectric nanogenerator	Micromolding	Delivery of LevCDs/ES	(118)
	PCLMA/HAMA/thiolated heparin	3D printing	Indication of infection/smart delivery of minocycline hydrochloride	(119)
	MeHA/DMOG/ZIF-8	Micromolding	Antibacterial effect/angiogenesis	(61)
	PEGDA/CMCS/Cu@ZIF-8	Micromolding	Antibacterial effect/angiogenesis	(152)
	SilMA/MOF/curcumin/PVA	Micromolding	Wound pH analysis/rapid self-sterilization	(120)
	CS/NIPAM	Micromolding	Antibacterial effect/smart delivery of VEGF	(81)
	CS/fucoidan/kangfuxin	Micromolding	Delivery of kangfuxin	(153)
	HA/PVA/S-nitrosoglutathione/GO	Micromolding	Smart release of NO	(154)
	Arginine/CS/PVA	Micromolding	Delivery of curcumin	(56)
*Diabetic wound healing*	PVA/GelMA/black phosphorus/hemoglobin	Micromolding	Responsive oxygen release	(124)
	poly-γ-glutamic acid/graphene oxide-silver nanocomposite/Mg-MOF	Micromolding	Antibacterial effect/release of Mg^2+^ and gallic acid	(125)
	GelMA/SilMA	Micromolding	Release of AgNPs and MSC-derived exosomes	(126)
	MeHA/Fe_3_O_4_/lipoic acid sodium	Micromolding	Delivery of SeNPs/magnetic field-based therapy	(123)
	SF/NIPAM/SiO_2_ NPs	Micromolding	Biochemical analysis of inflammatory factors/delivery of VEGF	(127)
	SF/PU/MXene/NIPAM/SiO_2_ NPs	Micromolding	Controlled release of hEGF/biochemical analysis	(86)
	PVA/3 M detachable medical tape	3D printing	Delivery of MSC-exosomes/adjustable PVA hydrogel needle tips	(128)
	GelMA/PEGDA	Micromolding	To encapsulate adipose-derived stem cells and secrete growth factors	(129)
	CS/PVP	Micromolding	Delivery of Mg^2+^ and Panax notoginseng saponins	(155)
	HA/HAMA	Micromolding	Delivery of Fe-MSC-NVs and PDA NPs	(156)
	GelMA/PEGDA/β-CD-AOI_2_	Micromolding	Controlled release of exosomes and tazarotene	(157)
	GelMA/AFPBA	Micromolding	Responsive release of gluconic insulin	(70)
	MXene/poly-γ-glutamic acid	Micromolding	Delivery of asiaticoside	(158)
	HA/CS/SF	Micromolding	Delivery of tetracycline hydrochloride and deferoxamine	(159)
	Gelatin/polylysine/Fe^III^TA NPs	Micromolding	Release of NO/antibacterial effect	(160)
	PVA/GelMA/*Chlorella vulgaris*	Micromolding	Release of oxygen, Zn^2+^, and Mg^2+^	(72)
	PCL/PVP/polyacrylamide/PDA/Cu^2+^	Micromolding	Delivery of CaO_2_–HA NPs and metformin	(161)
	PVA/Gelatin/methacrylate anhydride	Micromolding	Delivery of α-amylase, M@G, and pro-angiogenesis peptide QHREDGS	(162)
	HAMA/CMCS/sodium alginate	Micromolding	Delivery of Antisense yycF-loaded GO and basic fibroblast growth factor	(163)
	HAMA/PDA/PVA	Micromolding	Delivery of MEs/mild heat	(164)
	Gelatin/HA	Micromolding	Delivery of tetracycline hydrochloride and hEGF	(165)
	PCL/dopamine-modified HA	Micromolding	Delivery of Doxycycline hydrochloride and deferoxamine	(166)
	CaO_2_/catalase/SilMA/AgNPs	Micromolding	Release of oxygen/ROS scavenging/antibacterial effect	(67)
	Taurine/PBzymes/HAMA	Micromolding	Delivery of taurine-loaded PBzymes	(167)
*Scarless wound healing*	SF/PVA	Micromolding	Physical intervention in the formation of scars	(139)
	Gelatin	Micromolding	Release of gallic acid and quercetin	(168)
	SilMA/dissolvable dextran	Micromolding	Release of bismuth nanosheets and verteporfin	(140)

a Abbreviations: HA, hyaluronic acid; SF, skin fibroin; PEGDA, poly(ethylene glycol) diacrylate; ES, electrical stimulation; PLGA, poly(lactic-*co*-glycolic acid); GelMA, gelatin methacryloyl; MSC, mesenchymal stem cell; HAMA, hyaluronic acid methacryloyl; VEGF, vascular endothelial growth factor; PU, polyurethane; NIPAM, *N*-isopropylacrylamide; NP, nanoparticle; SP, spidroin; EGaIn, eutectic Galium-Indium; hEGF, human epidermal growth factor; PBA, 3-(acrylamido)phenylboronic acid; CS, chitosan; PVA, poly vinyl alcohol; LevCDs, covalent bonding of levofloxacin and carbon quantum dots; PCLMA, methacrylated polycaprolactone; MeHA, methacrylated hyaluronic acid; DMOG, dimethyloxalylglycine; ZIF-8, zeolitic imidazolate framework-8; CMCS, carboxymethyl chitosan; SilMA, silk fibroin methacryloyl; MOF, metal–organic framework; GO, graphene oxide; NO, nitric oxide; PDA, polydopamine; AFPBA, 4-(2-acrylamidoethylcarbamoyl)-3-fluorophenylboronic acid; PCL, polycaprolactone; PVP, poly(vinylpyrrolidone); CaO_2_–HA, sodium hyaluronate modified CaO_2_; M@G, glucose oxidase loaded MIL-101; ME, M2 macrophage-derived exosome; PBzyme, Prussian Blue nanoscale enzyme.

### 4.1 Acute Wound Healing

**Figure 4:**
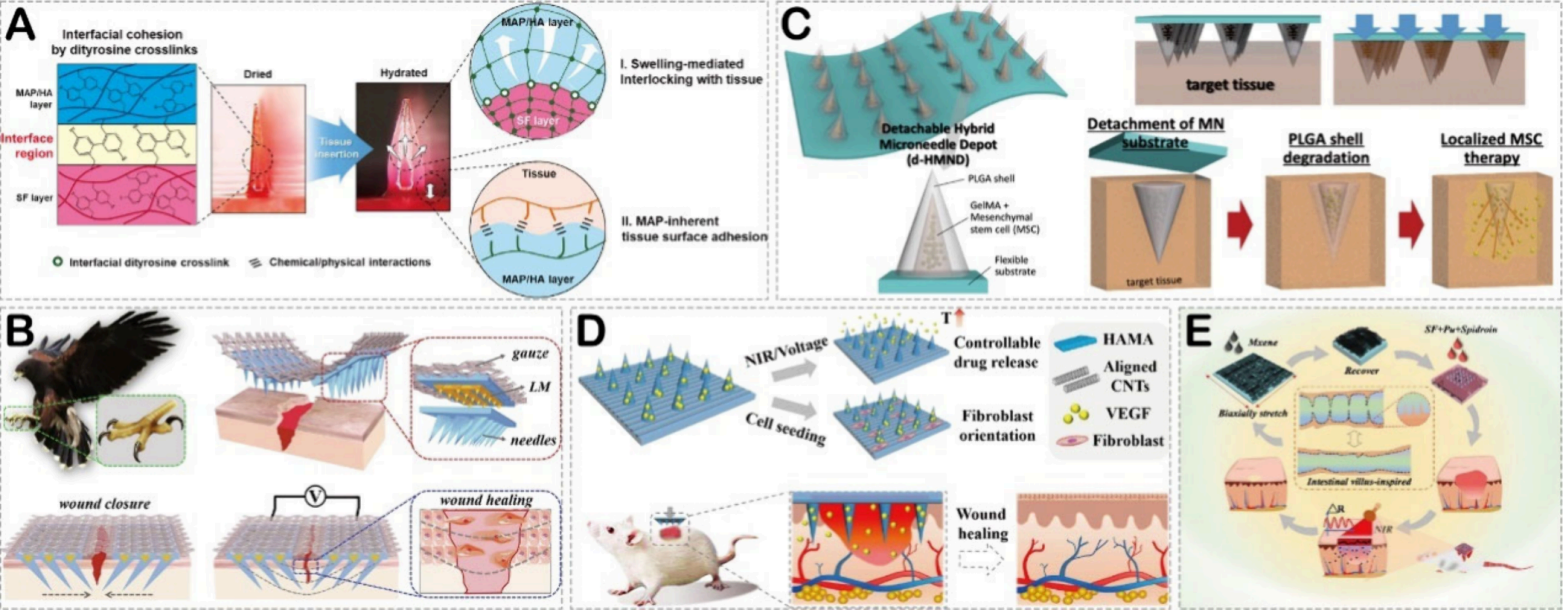
(A) The working mechanism of the adhesive HMN patch composed of a nonswellable SF-based core and a swellable and sticky MAP-based shell. Reproduced with permission from ref (110). Copyright 2019 Elsevier. (B) Illustration of the claw-shaped microneedle patch encapsulated with LM and its application in wound healing. Reproduced with permission from ref (85). Copyright 2020 Elsevier. (C) Schematic illustration of the assembled d-HMND loaded with MSCs and its application in wound healing. Reproduced with permission from ref (111). Copyright 2020 Wiley. (D) The working mechanism of the aligned CNT layer basement and the application of the multifunctional microneedle patch in wound healing. Reproduced with permission from ref (112). Copyright 2022 Elsevier. (E) Illustration of the fabrication of intestinal villus-inspired microneedle dressing with MXene coating and its application in wound healing. Reproduced with permission from ref (113). Copyright 2023 Elsevier.

Acute wounds are typically caused by trauma or surgery and have predictable tissue repair processes. The key to treating acute wounds is to clean the wound and suture it to reduce bleeding, prevent wound dehiscence, and minimize external bacterial infections. The current clinical method for wound closure is surgical suturing, but it may result in tissue damage, scar formation, and intense inflammatory reactions.^109^ Therefore, it is necessary to develop alternatives for wound closure. Utilizing HMNs with adhesive properties or special structures to close wounds is a viable strategy for accelerating the healing of acute wounds. For example, inspired by endoparasites that swell their proboscis to anchor to the host’s intestines, Jeon et al.^110^ designed a double-layer HMN patch (Figure 4A). The patch achieved wound sealing by utilizing both surface adhesion and physical entanglement. Its structure consisted of an Expan swellable dable outer shell made of mussel adhesive proteins and a nonswellable inner core made of SF. Once the patch was inserted into the skin, the swellable microneedle tips mechanically interlocked with the tissue, and the strong adhesive properties of mussel adhesive proteins enabled the patch to firmly adhere to the wound tissue, successfully sealing the wound. In a rat model with a long incision wound, the use of this patch significantly accelerated wound healing without leaving scars, outperforming traditional surgical suturing methods. Similarly, inspired by eagle claws, Zhang et al.^85^ developed a liquid metal (LM)-encapsulated HMN patch (Figure 4B). The patch consisted of a breathable mesh connecting two inclined Poly(ethylene glycol) diacrylate (PEGDA) hydrogel sections, with LM embedded and connected to each microneedle. The tips of the two HMNs were inclined, forming a claw-like gripping structure that could grasp the skin near the wound and prevent wound dehiscence. Additionally, by connecting an external power supply to the LM, a spatial electric field could be generated to continuously apply electrical stimulation (() to the wound, thereby promoting the healing process. In vivo experiments demonstrated that this HMN patch exhibited excellent efficacy in treating acute wounds in rats.

Delivering stem cells is another important strategy for HMNs to accelerate the healing of acute wounds. For example, Lee et al.^111^ proposed a detachable hybrid microneedle depot (d-HMND) for delivering mesenchymal stem cells (MSCs) to promote wound healing (Figure 4C). The outer layer of the HMND was made of poly(lactic-*co*-glycolic) acid (PLGA), while the inner layer consisted of a mixture of MSCs and GelMA. GelMA hydrogel was suitable for maintaining the viability of MSCs, while the PLGA shell served a protective role and provided the mechanical strength required for insertion into the target tissue. In a mouse model with full-thickness skin defects, animals treated with d-HMND exhibited higher wound closure rates, improved reepithelialization, and increased vascular regeneration. There are also studies that utilize HMNs for delivering growth factors such as VEGF and human epidermal growth factor (hEGF) to accelerate wound healing. For instance, Sun et al.^112^ designed a multifunctional HMN patch composed of carbon nanotubes (CNTs) and hyaluronic acid methacryloyl (HAMA), which encapsulated VEGF (Figure 4D). The ordered structure of the CNT-based substrate not only imparted shape characteristics to the patch but also enabled controlled release of VEGF through its photothermal or electrothermal conversion ability. Further research demonstrated that the ordered microstructure of CNTs effectively induced the alignment of fibroblasts, while the release of VEGF promoted the tube formation of endothelial cells, significantly accelerating the healing of acute wounds in animal experiments. Inspired by the intestinal wrinkles and villi structure, Lu et al.^113^ constructed a multifunctional HMN dressing based on MXene, SF, and polyurethane (Figure 4E). Due to the photothermal responsive properties of MXene, this system allowed for controlled drug delivery through near-infrared (NIR) radiation. In a mouse model of acute wounds, researchers successfully accelerated wound healing by delivering hEGF using this HMN. Additionally, there are studies reporting the use of HMNs for delivering adenosine and asiatic acid to accelerate the healing of acute wounds.^114,115^ Further information can be found in Table 2.

### 4.2 Infected Wound Healing

Bacterial infection has always been one of the main causes of delayed wound healing, affecting millions of patients every year.^116^ However, traditional antimicrobial methods have certain limitations. For instance, systemic use of antimicrobial drugs lacks specificity and often causes severe side effects. Antibiotic ointments or creams can only target surface infections and cannot penetrate deep into tissues. Subcutaneous injections are painful and patients often exhibit poor compliance. Moreover, clinical treatment of bacteria protected by biofilms is particularly challenging.^117^ HMNs possess excellent biocompatibility and can easily disrupt bacterial biofilms, delivering antimicrobial agents to deeper tissues without the side effects associated with systemic administration. Therefore, there is increasing research focus on the application of HMNs in infected wounds.

**Figure 5:**
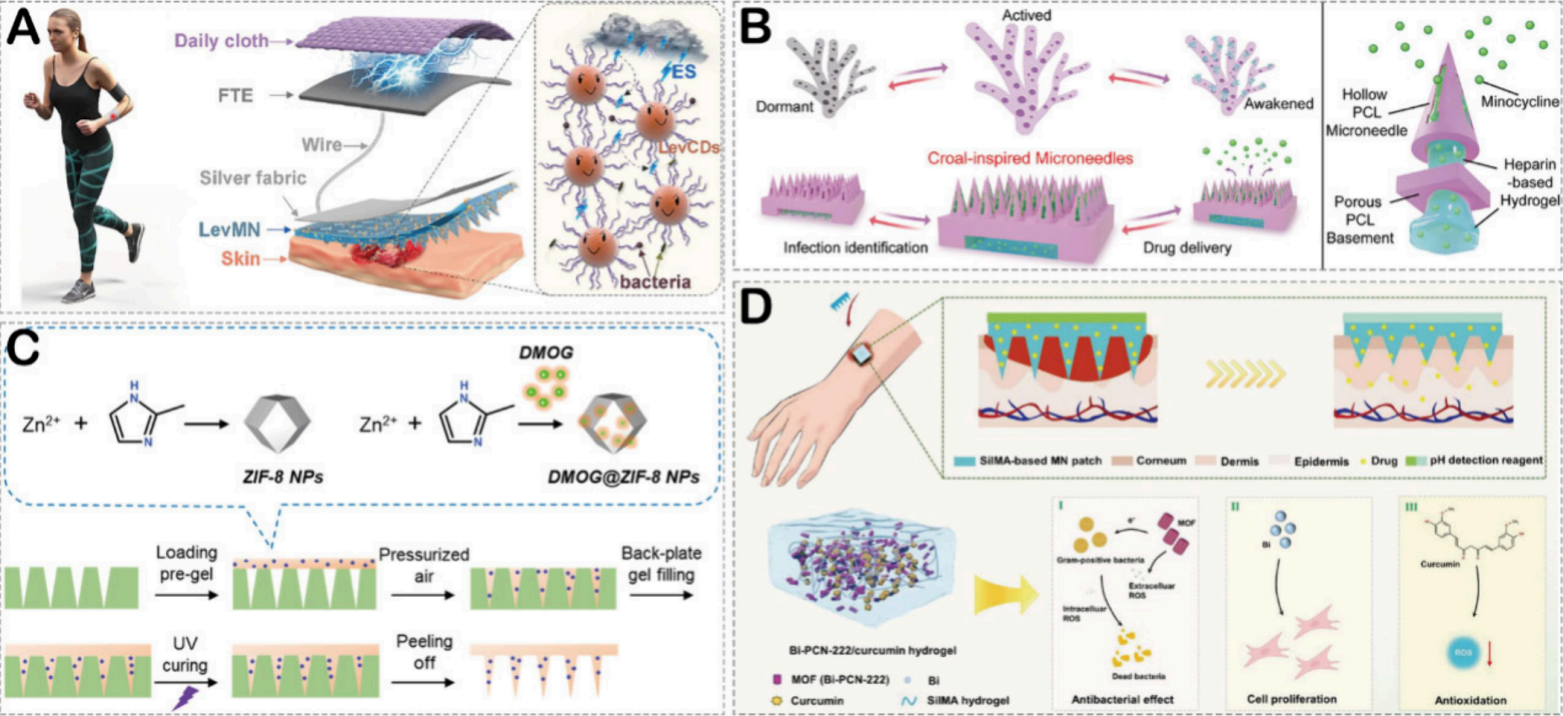
(A) The LevCDs-loaded microneedle patch for accelerating the healing of infected wounds. Reproduced with permission from ref (118). Copyright 2023 Elsevier. (B) Schematic diagram of coral-inspired microneedle patch. Reproduced with permission from ref (119). Copyright 2024 Wiley. (C) Schematic illustration of the preparation of HMN loaded with DMOG@ZIF-8. Reproduced with permission from ref (61). Copyright 2023 American Chemical Society. (D) HMNs with Bi-PCN-222 encapsulation for the treatment of infected wounds. Reproduced with permission from ref (120). Copyright 2024 Wiley.

Antibiotics are the foundation of modern medicine, as their use can kill bacteria and reduce infection-related mortality. The delivery of antibiotics for the treatment of wound infections through HMNs has been widely reported. For instance, Zhang et al.^118^ synthesized a novel conductive drug, LevCDs, through covalent bonding of levofloxacin and carbon quantum dots (Figure 5A). They prepared HMN patches using the PEGDA-enhanced classical PAAm-alginate composition, loaded with LevCDs and triboelectric nanogenerators. These LevCDs-loaded microneedle patches exhibited excellent antimicrobial performance, effectively eradicating bacterial colonies under ES. Furthermore, the ES promoted cell migration and proliferation, accelerating the healing of infected wounds in mice. Inspired by corals, Liu et al.^119^ developed a biomimetic HMN patch, which was called HepMi-PCL, for intelligent delivery of minocycline hydrochloride (Mi) (Figure 5B). They utilized high-precision 3D printing to fabricate polycaprolactone (PCL) microneedles with hollow porous structures of the same size. Subsequently, a thiolated heparin-methacryloylated hyaluronic acid hydrogel (HepMi) loaded with phenol red and Mi was filled into the PCL cavities, resulting in the HepMi-PCL smart microneedles. Upon absorbing infected exudate, HepMi-PCL intelligently activated to release Mi, thereby achieving antimicrobial functionality. As the infection subsided and exudate decreased, the drug release correspondingly slowed down or ceased. Additionally, it could collect exudate from wound tissue and indicate infection through a pH-induced colorimetric response based on phenol red. This microneedle system demonstrated significant potential in the treatment of infected wounds in rats, enhancing wound healing by over 200%.

Metal–organic frameworks (MOFs) are considered promising antimicrobial materials. They can act as carriers to encapsulate bioactive agents, thereby inhibiting bacterial growth.^120^ Additionally, they can serve as enzyme-like materials with excellent antimicrobial properties for infected wound healing.^121^ In recent years, research on the encapsulation of MOFs in HMNs has been widely reported. As mentioned above, Qin et al.^61^ prepared novel functional HMN patches for the treatment of infected wounds by loading pH-responsive functionalized ZIF-8 into MeHA (Figure 5C). The patch effectively killed bacteria by releasing Zn ions. Furthermore, dimethyloxalylglycine (DMOG) within the ZIF-8 framework could enhance angiogenesis in the wound bed by upregulating the expression of HIF-1α, thereby accelerating the healing of infected wounds. Xiao et al.^120^ utilized HMNs with Bi-PCN-222 encapsulation for the treatment of infected wounds (Figure 5D). They incorporated Bi-PCN-222 and curcumin into SF-methacryloyl (SilMA) hydrogels, resulting in HMN patches with multiple functionalities. Bi-PCN-222 eliminated bacteria by transferring electrons from itself to interfere with the metabolism of *Staphylococcus aureus*, while curcumin exerted anti-inflammatory effects, synergistically promoting the healing of infected wounds. Additionally, PVA hydrogel loaded with pH-sensitive fluorescent indicators served as the microneedle patch substrate, allowing real-time monitoring of wound pH.

The key of treating infected wounds lies in eradicating bacterial infections. Therefore, there have been studies on using HMNs to deliver antimicrobial polymers such as chitosan, antimicrobial molecules such as nitric oxide, and Kangfuxin for the treatment of infected wounds. Table 2 offers a comprehensive overview of the more intricate details.

### 4.3 Diabetic wound healing

The incidence of diabetic wounds is high, and their treatment poses significant challenges, placing a heavy burden on the healthcare system. Due to complex factors such as persistent inflammatory responses, abnormal proliferation of epithelial cells, and bacterial infections, the healing of diabetic wounds is exceedingly difficult.^122^ Currently, treatment methods for diabetic wounds encompass negative pressure wound therapy, growth factor therapy, stem cell therapy, and autologous skin grafting, etc.^123^ Nevertheless, factors such as cost, pain level, and efficacy limit the widespread adoption of these treatment techniques. Therefore, the scientific community has been dedicated to developing cost-effective, safe, and effective methods for the treatment of diabetic wounds.

The elevated glucose levels in diabetic wounds promote bacterial growth and biofilm formation, further impeding wound healing. HMNs can overcome the physical barrier of biofilms and uniformly deliver antimicrobial agents to the interior of bacterial colonies, thereby exerting effective antimicrobial effects. Additionally, HMNs can efficiently deliver drugs that promote vascular and tissue regeneration, thus accelerating wound healing. Therefore, HMNs have emerged as a promising therapeutic approach for diabetic wounds. In recent years, increasing research has focused on utilizing HMNs to deliver critical substances such as oxygen,^124^ metal ions,^125^ NPs,^123,126^ growth factors,^86,127^ exosomes,^126,128^ and stem cells^129^ to enhance the healing of diabetic wounds.

**Figure 6:**
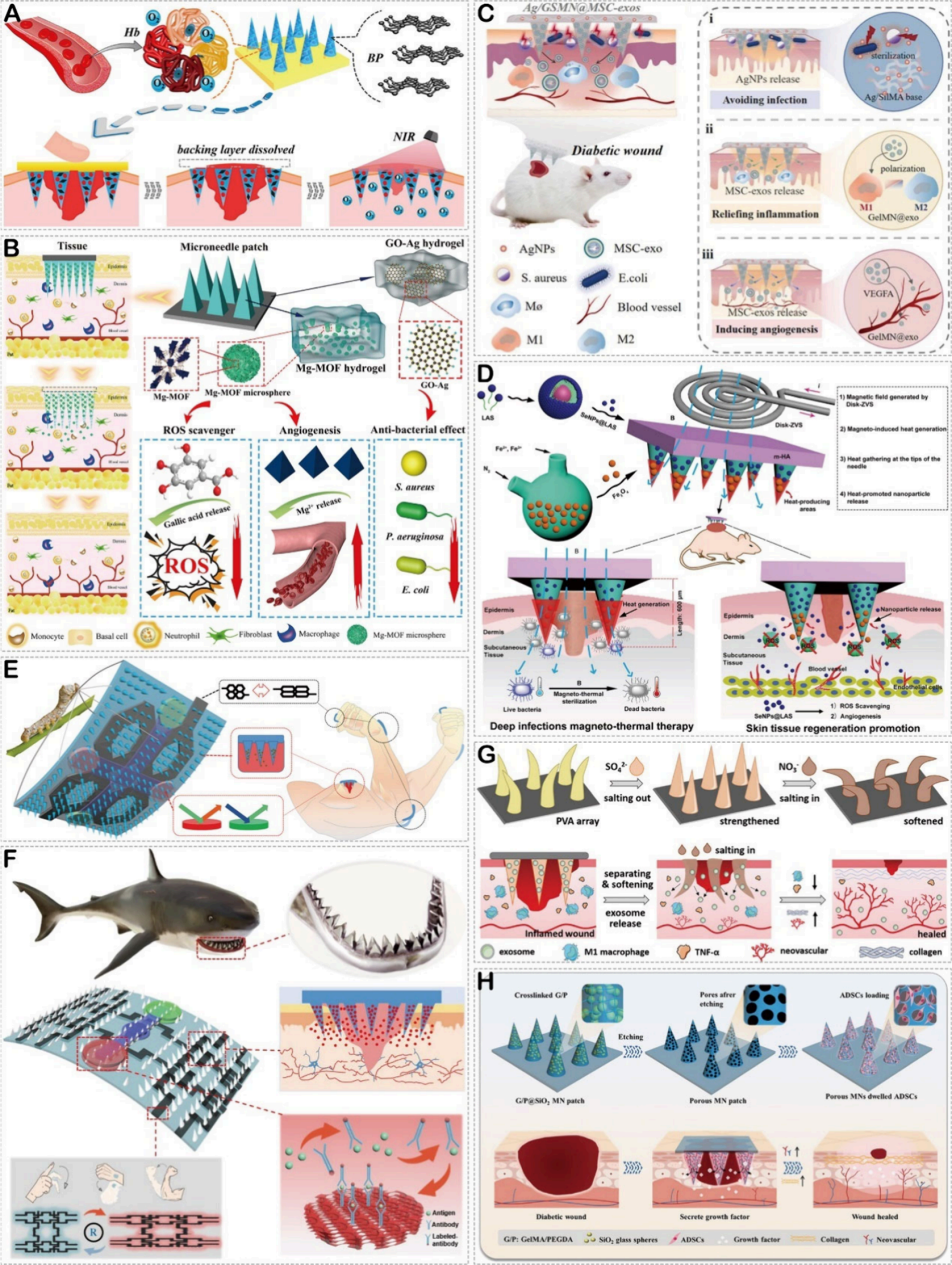
(A) Illustration of NIR-responsive separable HMN with BP QDs and hemoglobin encapsulation and its application in diabetic wound healing. Reproduced with permission from ref (124). Copyright 2020 American Chemical Society. (B) Schematic illustration of the Mg-MOF based HMN patch for accelerating diabetic wound healing. Reproduced with permission from ref (125). Copyright 2021 American Chemical Society. (C) Illustration of the adhesive HMN patch encapsulated with MSC-exos and antibacterial AgNPs for improving diabetic wound healing. Reproduced with permission from ref (126). Copyright 2022 Elsevier. (D) Schematic diagram of the construction of the HMN encapsulated with SeNPs@LAS and Fe_3_O_4_, and its magneto-thermal therapy for infected diabetic wounds. Reproduced with permission from ref (123). Copyright 2023 Wiley. (E) Schematic illustration of the intelligent i-SMD for biochemical sensing, motion monitoring, and wound healing. Reproduced with permission from ref (127). Copyright 2020 Wiley. (F) Schematic illustration of shark tooth-inspired microneedle dressing for intelligent wound management including motion sensing, biochemical analysis, and healing. Reproduced with permission from ref (86). Copyright 2021 American Chemical Society. (G) Schematic illustration of the adaptive mechanical strengths and wound healing mechanisms of microneedles composed of MSC-exos- encapsulated adjustable PVA hydrogel needles and detachable 3 M medical tape support substrate. Reproduced with permission from ref (128). Copyright 2023 Wiley. (H) Schematic diagram of the preparation of porous HMN loaded with ADSCs and its application in wound healing. Reproduced with permission from ref (129). Copyright 2024 Wiley.

Oxygen plays a crucial role in wound healing by promoting the survival of skin cells under hypoxic conditions and stimulating the production of growth factors necessary for wound repair.^130^ The delivery of oxygen through HMNs holds promise as a key strategy for treating diabetic wounds. For instance, Zhang et al.^124^ developed a responsive oxygen-releasing microneedle composed of a PVA backing layer and GelMA tips loaded with black phosphorus quantum dots (BP QDs) and hemoglobin (Figure 6A). Upon insertion into the skin, the PVA backing layer rapidly dissolved, while the GelMA tips remained in the wound. The GelMA tips, owing to the remarkable photothermal effect of BP QDs and the reversible oxygen-binding properties of hemoglobin, could achieve responsive oxygen release under NIR irradiation. Further animal experiments validated the potential of this microneedle system for treating full-thickness skin defects in type 1 diabetic rats.

Some metal ions such as Ag^+^, Zn^2+^, and Cu^2+^ possess antimicrobial properties and can effectively prevent the emergence of antibiotic resistance.^131^ Additionally, metal ions like Mg^2+^, Zn^2+^, and Cu^2+^ can promote cell proliferation and tissue regeneration.^132,133^ However, direct and repeated application of metal ions raised concerns regarding treatment safety. To address this issue, Yin et al.^125^ developed a double-layer HMN capable of slowly releasing Mg^2+^ and gallic acid (Figure 6B). In this microneedle system, the poly(γ-glutamic acid) hydrogel mixed with magnesium organic frameworks (Mg-MOFs) was used as the tips of the microneedle, while the poly(γ-glutamic acid) hydrogel loaded with graphene oxide-silver nanocomposites (GO-Ag) served as the backing layer. Mg-MOFs consisted of Mg^2+^ and gallic acid, which were slowly released under acidic conditions. The released Mg^2+^ exhibited low cytotoxicity, promoted angiogenesis, and regulated inflammation, while the ROS scavenger gallic acid exerted antioxidant functions. Moreover, GO-Ag in the backing layer showed excellent antibacterial effects. Furthermore, the researchers constructed full-thickness skin wounds in diabetic mice, and the results demonstrated a significant improvement in wound healing with the use of this microneedle treatment.

NPs have gained considerable attention in wound treatment as they can serve both as therapeutic agents and carriers for therapeutic drugs.^134^ An increasing number of studies focus on utilizing HMNs for the delivery of NPs in the treatment of diabetic wounds. For instance, Gan et al.^126^ designed an HMN capable of delivering antibacterial AgNPs and MSC-derived exosomes (MSC-exos) (Figure 6C). The SilMA hydrogel loaded with AgNPs was selected as the backing layer, effectively killing bacteria through the release of AgNPs. The microneedle tips were composed of GelMA hydrogel loaded with MSC-exos, which could slowly and continuously deliver anti-inflammatory and pro-angiogenic MSC-exos, thereby accelerating the healing process. This multifunctional microneedle patch demonstrated excellent therapeutic efficacy in treating full-thickness skin wounds in diabetic rats. Similarly, He et al. prepared a double-layer HMN capable of slow release of SeNPs (Figure 6D).^123^ In this microneedle system, cross-linked MeHA formed the structural framework, encapsulating two types of NPs: lipoic acid sodium (LAS)-protected SeNPs (SeNPs@LAS) in the base layer and Fe_3_O_4_ at the tips. As the GelMA hydrogel gradually degraded due to excess hyaluronidase in diabetic wounds, the release of SeNPs@LAS occurred, exerting antibacterial, anti-inflammatory, and pro-angiogenic effects of SeNPs. The Fe_3_O_4_ at the tips enabled magneto-thermal antibacterial functionality under the influence of the electromagnetic field. The researchers further created full-thickness skin defects infected with *Staphylococcus aureus* in diabetic mice and successfully utilized this slow-release SeNPs HMN to accelerate wound healing.

Growth factors produced during the wound healing process play a mediating and regulatory role in the angiogenesis and reepithelialization of skin wounds, promoting healing.^135^ An increasing number of studies focus on utilizing HMNs for the delivery of growth factors to promote diabetic wound healing. For example, Gao et al. designed an SF microneedle-structured dressing with a biochemical sensing and intelligent drug release system, called i-SMD, which could accelerate diabetic wound healing through the delivery of VEGF (Figure 6E).^127^ The researchers sequentially poured SiO_2_ solution and SF aqueous solution onto a PDMS mold, and after drying, they formed an inverse opal photonic crystals (IO PCs) structure in the SF microneedles through hydrofluoric acid etching. By pouring a temperature-responsive *N*-isopropylacrylamide hydrogel loaded with VEGF into the gaps of the IO PC structure, they successfully achieved controlled drug release on the i-SMD. Further animal experiments validated the potential of i-SMD for treating diabetic wounds. Additionally, they also developed a shark tooth-inspired biomimetic microneedle patch using a similar approach and accelerated diabetic wound healing by delivering hEGF (Figure 6F).^86^

MSC-exos have tremendous therapeutic potential as they can regulate cell proliferation and differentiation, thereby intervening in the entire healing process.^136^ Therefore, there are also studies dedicated to using HMNs to encapsulate exosomes for the treatment of diabetic wounds. As mentioned earlier, the HMN designed by Gan et al.^126^ could accelerate diabetic wound healing through the delivery of MSC-exos. In addition, Zhang et al.^128^ designed an adaptive indwelling HMN composed of PVA hydrogel tips encapsulating MSC-exos and detachable 3 M medical tape supporting substrate (Figure 6G). After insertion into the tissue, it could release MSC-exos. Due to the ability of MSC-exos to effectively activate fibroblasts, endothelial cells, and macrophages, this microneedle demonstrated a promoting effect on wound healing in a full-thickness skin wounds of diabetic rat models.

Stem cells can secrete various bioactive molecules, thereby regulating immune function and promoting tissue regeneration.^137^ Therefore, stem cell therapy is considered an advanced approach that propels wound treatment to a new stage. There are studies dedicated to utilizing HMNs encapsulating stem cells for the treatment of diabetic wounds. For example, Fan et al.^129^ designed an HMN encapsulating adipose-derived stem cells (ADSCs) for treatment of diabetic wound. They simply filled the negative mold with a mixture of GelMA/PEGDA containing glass microspheres and created a porous HMN array through overnight etching. Subsequently, they loaded ADSCs encapsulated in Matrigel into the MNAs through perfusion. Due to the porous structure of the HMN, ADSCs could absorb sufficient nutrients, proliferate extensively, and secrete porous cytokines that promoted wound healing. Moreover, the HMN exhibited mechanical strength that enabled effective penetration through the skin. Further rat experiments demonstrated that this ADSCs-loaded HMN could promote tissue regeneration and angiogenesis in diabetic wounds, making it a promising therapy for wound healing.

Chronic diabetic wounds have been a clinical challenge to treat, and as a result, a significant proportion of current research on HMNs for wound treatment is focused on diabetic wound therapy. Table 2 summarizes recent studies related to the treatment of diabetic wounds using HMNs.

### 4.4 Scarless Wound Healing

**Figure 7:**
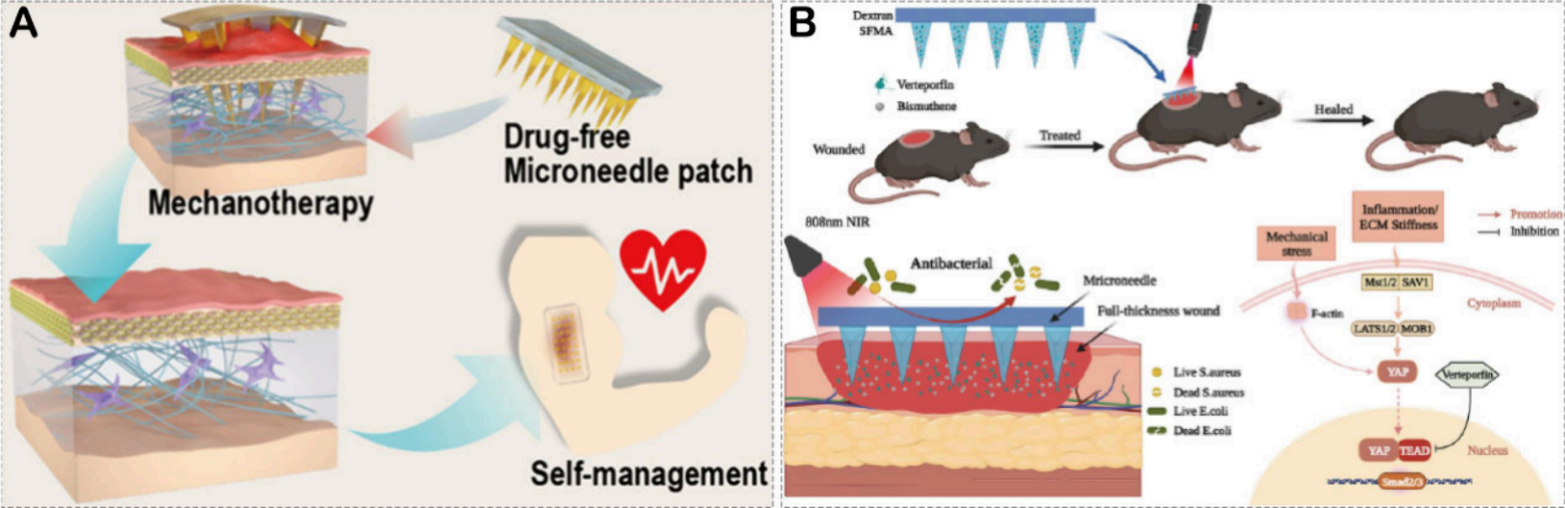
(A) Schematic diagram of the SF-based MNA patch for scar formation downregulation through the mechanical communication pathway. Reproduced with permission from ref (139). Copyright 2022 American Chemical Society. (B) Illustration of Bi/Vp@MN HMN patch for scarless wound healing. Reproduced with permission from ref (140). Copyright 2023 Elsevier.

The process of wound healing often accompanies scar formation, which can severely impact the physical and mental health of patients.^138^ Avoiding scar formation while promoting wound healing remains a significant challenge in the field of medicine. The use of HMNs as a mechanical therapeutic strategy or drug delivery system to interfere with the scar formation process holds promise for achieving scarless wound healing. For instance, Zhang et al.^139^ reported a method for scarless wound healing using a SF-based microneedle patch (Figure 7A). The researchers found that by adjusting the size and density of the microneedles, the biocompatible microneedles significantly reduced the scar elevation index in a rabbit ear hypertrophic scar model and increased the ultimate tensile strength close to normal skin. They further discovered that the SF-based microneedles attenuated integrin-FAK signaling, thereby downregulating the expression of TGF-β1, α-SMA, collagen I, and fibronectin. This created a low-stress microenvironment that contributed to a significant reduction in scar formation. This study demonstrates that microneedles, through a mechanical therapeutic strategy involving physical intervention, have the potential to achieve scarless wound healing. Wei et al.^140^ inhibited scar formation by delivering a YAP signaling pathway inhibitor, verteporfin, through HMNs (denoted as Bi/Vp@MN) (Figure 7B). They fabricated the SilMA hydrogel with loaded verteporfin and bismuth nanosheets into the MN tips, while dissolvable dextran was used to create the backing layer. Upon insertion into the skin, the backing layer of the HMN dissolved rapidly, and the SilMA tips remained in the wound area. The release of verteporfin inhibited scar formation by suppressing Yes-associated protein signaling, while bismuth nanosheets provided photothermal therapy for bacterial infections. Further animal studies indicated that this microneedle system could suppress excessive scar growth, making it a promising scarless wound healing approach.

Currently, there is relatively limited research on HMNs in the field of scarless wound healing. However, with the increasing demand for aesthetics, it is anticipated that more research will focus on this area in the future.

## 5 Conclusion and Outlook

Wound treatment, especially for chronic wounds, has always posed a significant challenge in clinical practice. However, wound dressings based on HMNs have garnered widespread attention as a potential solution. As polymeric microneedles with a three-dimensional network structure, HMNs exhibit excellent biocompatibility and biodegradability. Their insertion into the skin causes minimal discomfort, making them an ideal choice for drug delivery in wound treatment. Crucially, HMNs can carry various types of therapeutic drugs while maintaining their activity. Furthermore, drug delivery through HMNs directly targets the wound site, bypassing systemic circulation and hepatic metabolism, thus avoiding systemic drug toxicity.

Current research suggests that the use of HMNs holds potential benefits for wound repair. However, there is a lack of studies comparing the superiority of HMNs-based drug delivery to topical administration of the same therapeutic agents. Additionally, the impact of HMNs size and density on therapeutic outcomes has not been thoroughly investigated. Moreover, due to the relatively recent emergence of HMNs technology, there is a limited number of studies focusing on specific applications, such as wound repair. While numerous studies have emphasized the use of HMNs for chronic wounds, it is worth noting that the pathophysiological characteristics of the animal models used to assess treatment effectiveness, such as infected and diabetic wounds, are limited. Furthermore, most studies utilize small animals such as rats, mice, or rabbits, with only a few involving larger animals like beagles or pigs. It is important to acknowledge that HMNs are not a universal solution for wound treatment. In certain cases, particularly in wounds that necessitate extensive debridement, the use of HMNs alone may be insufficient to support wound repair. Although the small size of HMNs may reduce pain and tissue damage, it also limits their drug-carrying capacity and their ability to target deeper tissues. Therefore, in instances of wound infection combined with bone infection, more aggressive treatment measures may be necessary instead of relying on HMNs. Furthermore, the clinical application of HMNs must take into account immunogenicity concerns. Current research in this area primarily focuses on delivering various therapeutic substances through HMNs, including small molecules, macromolecules, NPs, exosomes, and stem cells. While stem cells can secrete multiple growth factors to promote wound healing, they may also present risks of immunogenicity and uncontrolled differentiation.

Despite the aforementioned limitations, the current research findings regarding the use of HMNs in wound treatment are promising. Further investigations are warranted to explore the application of HMNs in the field of wound repair, with a particular emphasis on expanding the range of delivered small molecules and engineered nanomaterials. For example, chemically modified fullerenes have exhibited favorable characteristics such as good water-solubility,^141^ improved biocompatibility,^142^ and photodynamic properties,^143^ which have made them widely utilized as photosensitizers in anticancer and antimicrobial photodynamic therapy. Furthermore, fullerenes possess excellent antioxidant,^144^ anti-inflammatory,^145^ and drug-loading capabilities,^146^ indicating the tremendous potential of fullerene-loaded therapeutic drugs delivered through HMNs in the treatment of chronic wounds, including infections and diabetes. Additionally, considering the biophysical properties of fullerene nanomaterials, including their ability to generate various ROS (e.g., singlet oxygen and superoxide anion radical), as well as their resistance to photobleaching, they appear to be an intriguing carbon-based nanomaterial with potential applications in microneedle technology.^147^ However, research specifically focused on this area has not yet been reported.

Furthermore, to obtain more comprehensive and accurate research results, it is necessary to expand the variety of experimental animal species and the selection of wound models. Pigs possess tissue structures highly similar to humans, particularly in terms of epithelial regeneration, making them ideal animals for constructing wound models.^148^ Therefore, utilizing pigs as experimental subjects would be of significant importance in studies focusing on wound repair assisted by HMNs. At the same time, it is necessary to construct more complex wound models, such as arterial/venous ulcers, pressure ulcers, and open wounds complicated by osteomyelitis.

Besides, given the differences in wound size, location, and required drug dosage among patients, customization is an essential direction for the future development of HMNs. 3D printing offers a viable solution for personalized customization by printing arbitrary structures based on 3D models obtained from patients’ wounds. However, this method of preparing HMNs is time-consuming and costly. In some cases, a single model may be suitable for a group of patients. Therefore, a combined application of 3D printing and micromolding may be necessary in the future to shorten the process and reduce costs. By utilizing 3D printing to produce the master mold for micromolding, HMNs can be manufactured in large quantities quickly through micromolding.

Moreover, intelligentization is also an inevitable trend for the future development of HMNs. Currently, the application of HMNs in wound management involves some smart responsive materials for simple wound monitoring or stimulus-responsive drug release. However, most of these smart materials can only detect a single indicator or respond to a single factor, such as ROS, pH, enzymes, or glucose, which cannot reflect the complex microenvironment of the wound and may be adverse to wound healing. The combination of HMNs and smart devices seems to provide a solution to this problem. HMNs first extract wound detection markers, and the sensors monitor and feedback information to the receiving device in real-time. The receiving device analyzes patient information, such as pH, ROS, and blood glucose content, and ultimately controls drug release. The combination of smart devices and HMNs will also facilitate more precise control of drug release rates and partially alleviate the problem of sudden drug release. There have been reports on the combined application of smart devices with HMNs for gastrointestinal drug delivery.^149^ With further in-depth research on HMNs and continuous advancements in smart devices, it is believed that HMNs can play an increasingly significant role in wound treatment by achieving intelligent regulation in terms of timing, location, and dosage according to the specific requirements of wound healing.
